# Validation of the WHO Hemoglobin Color Scale Method

**DOI:** 10.1155/2014/531670

**Published:** 2014-04-15

**Authors:** Leeniyagala Gamaralalage Thamal Darshana, Deepthi Inoka Uluwaduge

**Affiliations:** Medical Laboratory Sciences Unit, Department of Allied Health Sciences, Faculty of Medical Sciences, University of Sri Jayewardenepura, Gangodawila, Nugegoda 10250, Sri Lanka

## Abstract

This study was carried out to evaluate the diagnostic accuracy of WHO color scale in screening anemia during blood donor selection in Sri Lanka. A comparative cross-sectional study was conducted by the Medical Laboratory Sciences Unit of University of Sri Jayewardenepura in collaboration with National Blood Transfusion Centre, Sri Lanka. A total of 100 subjects participated in this study. Hemoglobin value of each participant was analyzed by both WHO color scale method and cyanmethemoglobin method. Bland-Altman plot was used to determine the agreement between the two methods. Sensitivity, specificity, predictive values, false positive, and negative rates were calculated. The sensitivity of the WHO color scale was very low. The highest sensitivity observed was 55.55% in hemoglobin concentrations >13.1 g/dL and the lowest was 28.57% in hemoglobin concentrations between 7.1 and 9.0 g/dL. The mean difference between the WHO color scale and the cyanmethemoglobin method was 0.2 g/dL (95% confidence interval; 3.2 g/dL above and 2.8 g/dL below). Even though the WHO color scale is an inexpensive and portable method for field studies, from the overall results in this study it is concluded that WHO color scale is an inaccurate method to screen anemia during blood donations.

## 1. Introduction

Anemia is a public health problem that affects both developed and developing countries. Pregnant women and young children are the most affected groups by its overwhelming effects. Hemoglobin concentration is considered as the most reliable indicator of anemia than the clinical findings [1]. The World Health Organization (WHO) color scale method is an inexpensive method for estimating hemoglobin concentration from a drop of blood by means of a color scale [2]. The color scale comprises a small card with six shades of red that represent hemoglobin levels at 4, 6, 8, 10, 12, and 14 g/dL, respectively [3]. Although many absorbent papers were tested, it was concluded that the Whatman 31 ET paper gave the best results with regular round stain of a limited spread. The color standards are printed in a continuous row without any separation and are mounted on a rigid white polyvinyl chloride or polypropylene sheet or thick card with a neutral pale-grey matt background. Estimation of the hemoglobin is done by matching the blood sample with the color standards through circular apertures which are placed in the center of each color standard [4]. The WHO color scale was primarily designed for anemia screening in obstetrical management, pediatric clinics, malaria and hookworm control programs, blood transfusion donor selection, and epidemiological surveys [2, 5, 6].

WHO color scale is a semiqualitative method and over the years it has been a useful tool in identifying anemia in field studies. Efficiency in terms of cost, accuracy, and time makes it an important resource in primary health care settings in developing countries. At present WHO color scale is the most widely used method for detecting anemia in settings where there is no laboratory. It performs better than clinical diagnosis alone in detecting mild to moderate anemia. However color scale's detecting ability is reduced as anemia becomes more severe [7].

Sensitivity and specificity of WHO color scale were very high in laboratory based studies but reduced considerably in field studies [7]. A comparative cross-sectional study done in Ethiopia showed a very low sensitivity in detecting anemia among pregnant mothers [5]. Sensitivity for the hemoglobin values <9 g/dL was 42.9% and for values <10 g/dL was 33.3% whereas sensitivity for the hemoglobin values <11 g/dL was 43.5%. However specificity remained relatively high in all three categories [5]. Underestimation of the high hemoglobin levels is also reported by Montresor et al. in a field study conducted to detect the anemia among preschool children in Zanzibar [6]. High number of false positives is another problem associated with the WHO color scale. Barduagni et al. have reported very low positive predictive value (PPV) for the color scale (26.7%) in a study which assessed the prevalence of anemia among school children in Northern Egypt suggesting that high number of healthy individuals can be labeled as anemic [8]. Similar results were reported by van den Broek et al. in a study assessing the potential of WHO color scale in anemia screening of pregnant mothers. Positive predictive values were very low for hemoglobin concentrations of ≤8 g/dL and ≤6 g/dL (11.1% and 15.8%, resp.) giving large amounts of false positives as anemic [9].

The predonation assessment of the blood donor hemoglobin is the best approach to determine the iron-status of the donor. Hemoglobin screening prior to blood donation is essential to safeguard anemic individuals from blood donating and protects returning donors from donation-induced iron deficiency [10]. WHO color scale is a common tool that is used to screen anemia during blood donation because of its simplicity [1]. However, some issues have been raised regarding its screening accuracy. Shahshahani and Amiri have reported relatively low sensitivity for the WHO color scale (54.5%) in a study which screened individuals prior to blood donation in Iran. Hemoglobin levels measured by color scale were significantly lower (0.32 ± 0.65 g/dL; *P* < 0.001) than the levels measured by the standard method [11]. In Sri Lanka too WHO color scale is the mostly used tool to screen anemia prior to blood donation. The present study was undertaken to evaluate the diagnostic accuracy of WHO color scale in screening anemia during blood donor selection in Sri Lanka.

## 2. Materials and Methods

A comparative cross-sectional study was conducted by the Medical Laboratory Sciences Unit of University of Sri Jayewardenepura in collaboration with National Blood Transfusion Centre, Sri Lanka. Study subjects were chosen from the donors who were attending above center and the data was collected between January and April 2010. Informed written consents were obtained from each and every participant prior to the inclusion. Ethical clearances were obtained in written statements form Ethical Review Committee of University of Sri Jayewardenepura and National Blood Transfusion Centre, Sri Lanka. A total of 100 subjects were selected as the participants for the study.

**Figure 1:**
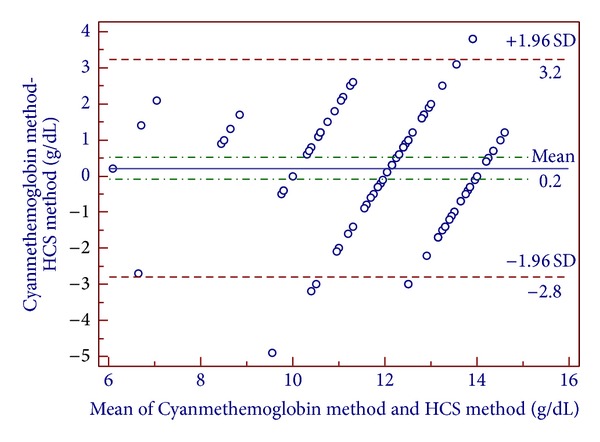
Bland-Altman plot for the haemoglobin colour scale compared with the reference method (cyanmethemoglobin method).

Finger pricked blood was used to measure the color scale hemoglobin value. A blood drop was placed on the test strip provided with the color scale and after waiting for 30 seconds the color of the blood spot was immediately matched against the given color standards (4, 6, 8, 10, 12, and 14 g/dL) and the corresponding value was recorded. Venous blood (2 mL) was collected from each subject into EDTA (ethylenediamine tetraacetic acid) containers for the laboratory assessments. Internationally recommended (gold standard) cyanmethemoglobin method was used to determine the reference hemoglobin concentrations of the blood samples [12]. Anticoagulated venous blood (20 *μ*L) was mixed with Drabkin's diluting fluid (5 mL) and after 5 minutes absorbance was taken at 540 nm by using a Labomed UV-VIS AUTO-UV-2602 spectrophotometer. Hemoglobin concentration was measured from a previously prepared standard curve with a hemoglobin standard (concentration of 660 mg/L to 250 times diluted blood of 16.5 g/dL). All the laboratory procedures including preparation of dilutions, absorbance reading, and measuring of hemoglobin concentration from the standard curve were done by single qualified laboratory technician to avoid the operator bias. Laboratory reference hemoglobin value was recorded in g/dL to one single decimal point and WHO color scale results were compared with the laboratory reference readings. Sensitivity, specificity, positive predictive value, and negative predictive values were measured. Bland-Altman plot and proximities of the color scale value to the reference value were obtained (Figure 1). All the statistical analyses were done by using Microsoft Office Excel 2007 and SPSS software version 12.0.

## 3. Results

Subjects were divided into five categories depending on their reference hemoglobin concentrations (Table 1). Sensitivity and the specificity of the WHO color scale remained low in all five categories; however, the sensitivity showed tendency to increase slightly when the hemoglobin concentration is increasing. Positive predictive value was very low in severe-moderate anemic regions (2.08%, in 5–7 g/dL; 4.44%, in 7.1–9 g/dL) indicating high rate of false positives at very low hemoglobin concentrations. Contrastingly, negative predictive value of the color scale remained relatively high in severe to mild anemic regions.

WHO color scale readings of 53 subjects out of 100 were within the range of the reference hemoglobin value ±1.0. Color scale results of the other 47 subjects were deviated from the reference value ±1.0. Seventeen subjects (17) had their hemoglobin values deviated from reference value ±2.0 (Table 2).

The mean difference value for the two methods (WHO color scale and cyanmethemoglobin method) was 0.2 g/dL. The limits of agreements for the two methods given by Bland-Altman plot were shown as the mean difference ±1.96 standard deviation. The limits of agreements for the WHO color scale were 2.8 g/dL below and 3.2 g/dL above.

## 4. Discussion

Sensitivity and specificity are two of the very important parameters required by a screening test to be validated. Diagnostic ability of a test method highly depends on these parameters. In the present study we observed low sensitivity and specificity values for all five hemoglobin concentrations (Table 1). Although slight increase in the sensitivity was observed when the hemoglobin concentration is increasing, that too was relatively low (55.55%) being the maximum sensitivity observed. The lowest sensitivity (28.57%) was observed in moderate anemic region (7.1–9 g/dL) and this result is somewhat similar to Gies et al. who have reported the lowest sensitivity (33.3%) of color scale for hemoglobin concentration <10 g/dL region indicating the poor accuracy of color scale in low hemoglobin concentrations [5].

In the present study, we observed low positive predictive values for the color scale. Lowest positive predictive value (2.08%) was observed in severe anemic (hemoglobin 5–7 g/dL) category. This implies high number of healthy nonanemic individuals can be diagnosed as anemic individuals. Our positive predictive value (4.44% for hemoglobin 7.1–9 g/dL) is even lower than van den Broek et al. who have reported positive predictive value (11.1%) for a similar hemoglobin range (≤8 g/dL) [9].

WHO color scale, at best, can measure hemoglobin value ±1 g/dL of reference hemoglobin value. Any value given by color scale outside this range would be inaccurate. In the present study only 53% of the data procured the appropriate range. Almost half of the values (47%) given by color scale being different from more than ±1 g/dL of reference value imply the poor performance of the color scale in field studies.

When examining the diagnostic accuracy of a test method (in this case WHO color scale) examining the agreement between test method and the gold standard method is vital. Bland-Altman plot was designed to measure the agreement and establish a limit of agreement of two test methods [13]. Therefore we used Bland-Altman plot to compare the agreement between the color scale and the reference instead of correlation coefficient or regression analysis. According to the results obtained from Bland-Altman plot the limits of agreement (the scattering area in which 95% of data are distributed) were 2.8 g/dL below and 3.2 g/dL above demonstrating a wide range of agreement (6 g/dL) for the color scale which is poor and unacceptable. The agreement would have been acceptable if it were 2 g/dL as ±1 g/dL change in color scale result to the reference value can be acceptable. Similar results were reported in a study done in England in which the limits of agreement for the WHO color scale were 3.50 g/dL below and 3.11 g/dL above and the range of agreement was slightly higher (6.61 g/dL) than the present study [14].

In the present study overall performance of the WHO color scale is not satisfactory. Interobserver variation could be a factor for the poor accuracy of the color scale. In this study color scale readings were taken by 3 public health inspectors who were working at the National Blood Transfusion Centre. Reading of the color scale under faded light or under weak light and the discoloration could be the factors interfering with the reading of color scale. Although it was made with Whatman 31 ET special chromatographic paper, there is a tendency to discoloration of the paper as it becomes older. This could substantially affect the reading of the color scale.

## 5. Conclusion

The WHO color scale is an inexpensive, portable, and easy method to screen anemia. Although its accuracy remains high in laboratory based studies, when it comes to field studies the accuracy becomes questionable. It was developed to be an alternative of the clinical evaluation of anemia and not of a spectrophotometer, but whenever a spectrophotometer is available that method should be preferred to the WHO color scale method in measuring the hemoglobin level. For the areas where spectrophotometers are not available clinical evaluation could be better than the WHO color scale. In future studies large sample numbers are recommended to obtain better results.

## Figures and Tables

**Table 1:** Reliability of WHO color scale with the reference method (cyanmethemoglobin method).

Hemoglobin concentration (g/dL)	5–7	7.1–9	9.1–11	11.1–13	>13.1
Specificity %	52.04	53.76	52.94	49.01	50.68
Sensitivity %	50	28.57	46.66	55.10	55.55
Positive prediction %	2.08	4.44	14.89	50.94	29.41
Negative prediction %	98.08	90.9	84.90	53.19	75.51

**Table 2:** Proximity of the test results to the reference method (cyanmethemoglobin method).

	Proximity to reference hemoglobin (g/dL)			
	±1.0	±1.1–2.0	±2.1–3.0	± >3.0
Number of subjects	53	30	12	05
